# Evaluation of tava program to improve the quality of life in young adults with autism spectrum disorders (ASD)

**DOI:** 10.1192/j.eurpsy.2021.1633

**Published:** 2021-08-13

**Authors:** A. Alvarez Pedrero, I. Parra Uribe, R. Muñoz, N. Reina, M. Llorens, A. Palacin, S. Membrives, J.A. Monreal, D. Palao Vidal

**Affiliations:** 1 Mental Health, Parc Taulí University Hospital. Autonomous University of Barcelona (UAB). I3PT, Sabadell, Spain; 2 Mental Health, Hospital Sant Joan de Deu., Barcelona, Spain; 3 Mental Health, Parc Hospitalari Martí i Julià, Girona, Spain; 4 Mental Health., Hospital Universitari Mútua de Terrassa. CIBERSAM, Terrassa, Spain

**Keywords:** autism, ASD, quality of life, Functionality

## Abstract

**Introduction:**

Autism spectrum disorders (ASD), is a population that does not usually receive specific treatment.

**Objectives:**

The main objective of the present study is to evaluate whether specific interventions within the TAVA program (transition program to adult life for patients with ADS), produce significant improvements in the quality of life of young adults with ASD.

**Methods:**

This is a prospective randomized clinical study of patients with ASD (according to DSM-5 criteria) seen in outpatient of the Parc Tauli University Hospital in Sabadell (Barcelona) since September 2017. We compared the quality of life, functionality, caregiver burden, and comorbidity of patients in TAVA program (beneficiaries of group therapy and specific medical and psychosocial interventions), with that of control patients (treatment as usual), after 2 years of intervention.

**Results:**

Our sample is composed of 12 patients with ASD. The average age is 18.4 years. 83% of the sample are men (n = 10). 5 of the patients belonged to TAVA and the other 7 were controls. Overall, TAVA patients presented improvement in the ZARIT and BAI scales compared to control patients. The control patients evolved less favorably in the AAA, SRS and RAAS levels compared to TAVA.

**Conclusions:**

Specific interventions in adults with ASD, improve the caregiver’s feeling of overload and the patients anxiety, compared to the usual interventions. The lack of regulated interventions produces an unfavorable evolution of the core symptoms of autism. More studies are needed to specify efficient interventions to improve the quality of life of adults with ASD.

**Disclosure:**

No significant relationships.

